# Why do – or don’t – patients with urinary tract infection participate in a clinical trial? A qualitative study in German family medicine

**DOI:** 10.3205/000221

**Published:** 2015-10-14

**Authors:** Jutta Bleidorn, Sermin Bucak, Ildikó Gágyor, Eva Hummers-Pradier, Marie-Luise Dierks

**Affiliations:** 1 Institute for General Practice, Hannover Medical School, Hannover, Germany; 2 Institute of General Practice and Family Medicine, University Medical Center, Goettingen, Germany; 3 Institute for Epidemiology, Social Medicine and Health Systems Research, Hannover Medical School, Hannover, Germany

**Keywords:** clinical trial, urinary tract infection, motivational factors, patients attitudes, trial participation, non participation, family medicine, qualitative research

## Abstract

**Background:** Insufficient patient recruitment can impair the conduct of clinical trials substantially, not least because a significant number of eligible patients decline trial participation. Though barriers and motivational factors have been worked out for patients with cancer or chronic diseases, little is known about primary care patients’ perceptions towards trial participation when visiting their family practitioner (FP) with acute uncomplicated conditions. This study aims to assess primary care patients’ motivation and barriers to participate in trials, and to identify factors that optimize patient recruitment in future trials.

**Methods:** This study was embedded in a drug trial comparing two treatment strategies for women with uncomplicated urinary tract infection in primary care. Semi-structured telephone interviews both with trial participants and decliners were conducted. The interview guideline focused on patients’ personal motivational or hampering factors. Further topics were study theme, FPs’ role, randomization, trial procedures, and potential motivational factors or barriers presumed to be relevant for other patients. Transcripts were analyzed by summarizing content analysis.

**Results: **20 interviews with trial participants and 5 interviews with trial decliners were conducted. Results show various reasons for trial participation from three categories: personal aspects, trial related aspects and patient-physician-relationship. A relevant trial topic and perceived personal benefit promotes participation as well as the wish to support research in general. Additionally, a maximum of safety concerning symptom relief reassures patients significantly. Trust in the FP plays also an important role in the decision process. Trial decliners show strong individual treatment preferences, which, together with individual reasons, lead to trial refusals.

**Conclusions: **To optimize recruitment conditions for further clinical trials on acute and common conditions in family medicine, the following key issues should be considered: emphasizing patients’ personal benefit, featuring patient relevant trial topics, providing a maximum of safety, keeping effort by trial procedures comfortable.

## Zusammenfassung

**Hintergrund:** Die Durchführung vieler klinischer Studien wird beeinträchtigt durch ungenügenden Patienteneinschluss, nicht zuletzt aufgrund einer nennenswerten Anzahl infrage kommender Patienten, die eine Studienteilnahme ablehnen. Auch wenn Barrieren und motivierende Faktoren für eine Studienteilnahme von Patienten mit onkologischen Erkrankungen oder chronischen Krankheiten bereits untersucht wurden, ist wenig bekannt über die diesbezügliche Einstellung hausärztlicher Patienten, die mit einer unkomplizierten Erkrankung ihren Hausarzt aufsuchen. Die vorliegende Studie hat zum Ziel, Motivationsfaktoren und Barrieren hausärztlicher Patienten für eine Studienteilnahme zu untersuchen, und Faktoren zu identifizieren, die die Patientenrekrutierung in zukünftigen Studien erleichtern.

**Methode:** Diese Studie war eingebettet in eine Arzneimittelstudie, in der zwei Behandlungsstrategien bei Frauen mit unkompliziertem Harnwegsinfekt im hausärztlichen Setting untersucht wurden. Semistrukturierte telefonische Interviews wurden sowohl mit Studienteilnehmerinnen wie auch mit Ablehnerinnen durchgeführt. Der Interviewleitfaden fokussierte auf den persönlichen motivierenden oder hinderlichen Faktoren der Patientinnen. Weitere Aspekte umfassten das Studienthema, die Rolle des Hausarztes, Randomisierung, Studienabläufe und potentielle motivierende/hinderliche Faktoren für andere Patientinnen. Die Analyse der Transskripte erfolgte mittels zusammenfassender qualitativer Inhaltsanalyse.

**Ergebnisse:** 20 Interviews mit Studienteilnehmerinnen und 5 Interviews mit Ablehnerinnen wurden durchgeführt. Die Ergebnisse zeigen unterschiedliche Gründe für eine Studienteilnahme aus den drei Bereichen: persönliche Aspekte, studienbezogene Aspekte und Beziehung zum Hausarzt. Ein relevantes Studienthema und ein wahrgenommener persönlicher Benefit fördert die Studienteilnahme ebenso wie der generelle Wunsch, Forschung zu unterstützen. Die Gewissheit, dass in jedem Fall mit einer Symptomlinderung zu rechnen ist, trägt wesentlich zur Beruhigung der Patientinnen bei. Auch das Vertrauen zum Hausarzt spielt eine wichtige Rolle im Entscheidungsprozess. Studienablehnerinnen äußerten deutliche Präferenzen hinsichtlich der Behandlung, was zusammen mit individuellen Gründen zur Ablehnung führte.

**Fazit: **Um die Rekrutierungsbedingungen für zukünftige klinische Studien zu akuten Erkrankungen im hausärztlichen Setting zu optimieren, sollten die folgenden Schlüsselaspekte berücksichtig werden: Betonung des persönlichen Benefits für die Patienten, Auswahl patientenrelevanter Studienthemen, Gewährleistung maximaler Sicherheit, geringer Aufwand durch Studienprozeduren.

## Background

Adequate patient recruitment is known to be crucial for successful clinical research in all fields of medicine. Until now, various methods to improve patient recruitment into trials have been described, e.g. telephone reminders, open-trial designs, opt-out strategies and financial incentives [1]. Nevertheless, more than a few trials fail due to inadequate patient recruitment, resulting in high costs for trial extension, or underpowered trials with high risk of bias [2], [3]. In many cases, both trial coordinators and investigators tend to overestimate the number of eligible patients [4]. Even though this may partly be due to patients with unexpected exclusion criteria, it is well known that a substantial number of eligible patients decline trial participation [5]. 

Thus, the “patient factor” leading to trial consent or refusal needs to be considered further. Both motivational factors and barriers for patients’ participation in trials have been worked out in many studies. The decision for trial participation seems to be complex and driven by various factors, summarized as a “personal balance account” by Verheggen et al. [6]. Altruism, the wish to support research and to help other affected patients, is often discussed as an important motivational factor [7], [8]. Many patients may perceive a moral obligation “to do a service to the community of sufferers to whom they belong” [9], [10], [11]. Further, confidence and trust in the doctor are described as promoting factors in various trials [11], [12]. 

Additionally, patients’ self-interest gains in importance. Canvin et al. subsume drivers for trial participation as “weak altruism with maintaining self-interest” [9]. More recently, the perceived benefits and the weighing up between benefit and disadvantages are discussed. McCann et al. describe this in their meta-review as an interaction of situational factors, patients’ view about the trial, personal factors and weighing of benefits [13], [14], [15]. On the other hand, in patients declining trial participation individual preferences and perceived disadvantages from trial treatment, e.g. from randomization without treatment choice, seem to have more weight [11].

Yet, most of these studies are based on patients with cancer or chronic conditions, being recruited during hospitalization, from specialists’ clinics or trial walk-in clinics. To our knowledge, there is little evidence on trial participation of patients with acute and uncomplicated conditions, treated and eventually enrolled in trials in family practices. Since clinical research grows in importance particularly in common conditions responsible for a large number of prescriptions, these otherwise healthy patients with acute conditions need special consideration as potential trial participants.

To optimize prerequisites for further clinical trials, we assessed these patients’ motivation and barriers to participate in trials by performing an embedded interview study in a double blind RCT comparing two treatment strategies in uncomplicated urinary tract infection (UTI) in German family practices [16]. This study aimed

- to assess primary health care patients’ motivation and barriers to participate in trials.
- to identify relevant benefits and barriers to be used for information and motivation of future trial participants.

## Methods

To capture a broad impression of patients’ motivational factors and barriers for trial participation, we performed this interview study with patients affected by an uncomplicated UTI who took part or declined participation in the clinical drug trial ICUTI.

### Urinary tract infection/description of the ICUTI trial

Uncomplicated UTI is a common, acute condition in women, associated with bothersome symptoms like painful voiding, urgency and abdominal pain [17] and usually treated with antibiotics according to current guidelines [18]. However, many affected women try to treat themselves with teas, herbal remedies or homeopathic products before consulting their family practitioner (FP).

To assess whether anti-inflammatory agents could be recommended as an alternative to antibiotic treatment for UTI, ICUTI was performed as a double blind, randomised controlled trial comparing immediate antibiotic treatment with fosfomycin (control group) to initial treatment with ibuprofen and conditional antibiotic treatment only if necessary (intervention group) [16]. ICUTI was conducted in 42 family practices in Lower Saxony and Bremen, and lead by the Departments of Family Medicine of Hannover Medical School and University Medical Center Goettingen. The entire trial was funded publically by the German Ministry of Research and Education (BMBF).

#### Trial Procedures

ICUTI participants were otherwise healthy women presenting with typical UTI symptoms. In all, 1,184 patients were assessed for egibility from February 2012 until February 2015. Of these, 405 did not meet inclusion criteria and 281 refused to participate. In four cases the reason was not specified. 494 patients were randomized to the two treatment arms, and 246 completed the study (finished follow up).

Usually, the practice assistant made UTI patients aware of the trial and delivered a ten-page ICH-GCP-compliant trial information sheet to be read in the waiting room. During consultation, the FP provided further information and obtained informed consent. The FP also handed out the blinded trial medication and instructed the patient to consult again in case of persistent or worsening symptoms. In this case, the trial drug could be discontinued and replaced by an antibiotic at the discretion of the FP. At inclusion, patients completed a symptom questionnaire to score severity of UTI symptoms. They received a diary to support symptom documentation during the following days, and an emergency card in case of medical emergencies requiring immediate unblinding even at night/during weekends.

Further symptom assessment took place via telephone interviews by study nurses of the University Departments’ research teams on day 1, 3, 5 and 7 or until symptom resolution. A final telephone interview took place on day 28. Participants received an expense allowance of 20 €.

### Interview partners and conduction

Interview partners were recruited as follows:

- Trial participants: From November 2012 until May 2013 and again in September/October 2013, study nurses of the Hannover trial team asked ICUTI patients during the final follow-up telephone interviews (day 28) for a further interview regarding trial participation. To avoid practice bias, maximally two patients per practice were asked for participation in the interview study. During the specified period 29 patients ‘eligible for interview’ had been enrolled in ICUTI. Of those, 25 were asked to participate in the interview study. In four cases the request was inadvertently forgotten. Two patients declined participation, and in three cases the arranged interviews could not be conducted. Patients who agreed to an interview were called again by an independent interviewer (SB) and were sent a study information and informed consent sheet. Upon receipt of the consent sheet, an interview date was arranged. All patient-related data were kept totally separated from ICUTI data.
- Decliners: To recruit patients who declined trial participation, we asked practice assistants and FPs in well recruiting ICUTI practices (n=8) to ask decliners to participate in the interview study. Agreeing patients received an information and consent sheet and left their phone number in the practice to be called by the interviewer (SB). Six decliners were recruited but one turned out to be not suitable for analysis since the FP has advised her against trial participation for medical reasons.

The semi-structured telephone interviews were then carried out by SB who was not involved in the ICUTI trial. All interviews were audio-recorded digitally and transcribed verbatim. The Ethics Committee of Hannover Medical School approved the embedded interview study.

### Interview guideline

A semi-structured interview guideline both for participants and decliners was developed including patients’ own motivational or hampering factors and further topics such as study theme, FPs’ or practice staff’s roles, blinding, and expense allowance. Initially, patients were asked to remember the situation of having been invited to participate in the ICUTI trial when visiting their FP with UTI symptoms, and to remember their reasons to participate or refuse, respectively. Further questions included possible motivational factors or barriers which could be important for other patients. The interview guideline had previously been discussed in the study team, and tested in two interviews.

### Analysis

Transcripts were analyzed by summarizing content analysis according to Mayring [19]. Data analysis was conducted independently by SB (Physician) and JB (MD, senior researcher, ICUTI coordinator) and was discussed with MLD (sociologist/psychologist, not involved in ICUTI). Initially the text was closely paraphrased; emerging topics were condensed to primary and secondary codes and matched to (sub) categories, stipulated by the guide and supplemented by new categories and subcategories. Below, they are indicated by headings, subheadings and underlining, and illustrated by original quotations. 

Transcripts of decliners are presented more narratively due to the small number.

## Results

In total, 20 interviews of trial participants (mean age 37 years) were analyzed, with a mean duration of 14.3 min. Additionally, five decliner interviews could be analyzed. For further details see Table 1 (Tab. 1). 

Results are divided into three sections: First, participants’ motivation and influencing factors are presented. Second, trial decliners’ reasons are shown, and third, barriers for trial participation from participants’ view are described. 

### 1) Participants’ motivation and influencing factors 

This section includes participating ICUTI patients’ motivational and influencing factors for trial participation. Many issues were addressed which were condensed in categories and subcategories. The main categories are a) personal aspects, b) trial-related aspects, and c) FP-patient-relationship.

#### a) Personal aspects

 In this category we subsume independent factors deriving from the patient herself, e.g. underlying character attributes for trial decision or personal experiences with research. 

**Altruism: **Basic character traits seemed to play a role in the decision process. Many interviewees showed a strong intention to **support research in general**. With or without UTI history, they considered it important to contribute to further development of medicine in general, knowing that this kind of research requires participating patients.

P6: “(…) without people who participate research just can’t be done. (…) if research is not done, then you can’t find out anything. And if no one participates in such studies, then that’s a problem.”

Others emphasized that they wanted to **help other women** affected by UTI to receive a better therapy. Perceiving antibiotics as bad and harmful, one patient showed a strong **sense of obligation to help others**, expressing this “help” clearly as a “protection from antibiotics”.

P3: “(...) you have to do your part so that such studies can be run. If there really is the possibility that you might protect patients from always having to take antibiotics, (…) then you have to do your part.”

**Spontaneity:** For some of the interviewees, the decision to take part in the trial was made intuitively. They rated themselves as curious, open-minded or spontaneous, and saw no need to think too long about the decision.

P14: “I spontaneously declared my readiness. As such, I have not given it much thought before. I was just being open and curious.”

**Research experiences** were also mentioned as motivational factors. One interviewee had experienced impressive personal benefit from former trial participation many years ago which made her take part again. Others knew the research setting from their work conditions, i.e. as a laboratory assistant.

P10: “(…) because I’ve already participated in such a study, I said, okay, this is for me ... if something like that is running, I’ll take part.”

#### b) Trial-related aspects 

This category includes aspects connected specifically to the ICUTI trial.

A **relevant trial theme** in terms of **avoiding unnecessary use of antibiotics** turned out to be an important factor. Strikingly, nearly all interviewed patients showed a critical attitude towards antibiotic therapy. Even if antibiotics were considered as important treatment opportunities, patients depreciated them as being prescribed too often, leading to bacterial resistances and suppressing the body’s own help mechanisms. Strong words were used to describe this, like “patients are pumped full of antibiotics”, “crammed with this stuff”, “chemical cosh”. 

Against this background, in particular patients with recurrent UTI and a history of antibiotic treatment for UTI expressed a strong interest for therapeutic alternatives.

P9: “(…) and since I suffer from this, I am also in favor that a solution for this is finally found. Not that I’m constantly crammed with antibiotics.”

One interviewee appreciated explicitly the aspect of patient-centered research in the ICUTI trial, contrasting to usual commercialized development in the health system.

P14: “(...) the whole health care system is more like an industry and ways to maximize profit are being looked for, so (...) that the individual is being forgotten. As such, I think it’s positive that in this [study], the person is still in the focus, which actually should be the case.”

A perceived **personal benefit** seemed to promote trial participation in many cases. In particular, patients affected by recurrent UTI described two main benefits for their own health: avoiding “bad and harmful” antibiotic treatment, and being able to handle future UTIs themselves without FP consultation.

P17: “(…) it has helped me. I would not immediately have antibiotics prescribed for me for a urinary tract infection.”

P8: “(...) for me, it was also the knowledge, that you don’t need to go to the doctor every time with a urinary tract infection, but rather that you can get rid of it yourself with ibuprofen over several days.”

The role of **material benefits** was assessed differently: Consistently, nearly all interviewees reported that the expense allowance had no importance for them when considering trial participation. 

However, the practical advantage to receive drugs immediately without pharmacy fees was appreciated.

P17: “Firstly the drugs were free, which I found good. And you did not have to pay five Euros [pharmacy charge], which was also a factor.”

**Trial safety:** The feeling of safety, expressed as “nothing (bad) can happen” was pointed out repeatedly as a decision-confirming factor. Some patients expressed this contrastingly – a lack of safety would mean a substantial personal disadvantage and a barrier for participation.

Safety included several aspects: Many interviewees mentioned that they felt reassured since they knew that **UTI is not a serious condition** – in case of which they had probably not agreed to participate in a trial.

P14: “I did not consider my illness as so serious that I had any concerns. If I really had something serious, with which they would then somehow have experimented on me, I would have probably not agreed.”

Furthermore, patients felt on the safe side since we used** well known drugs**, being in clinical use for a long time and known to be effective for pain from own experiences. In contrast, trials testing new drugs were perceived as risky, making the impression of “acting as a guinea pig” – with negative effect on willingness to participate in these trials.

P15: “This was a safe study for me. Since I knew: I would get an antibiotic effective against cystitis, or ibuprofen, which I know I tolerate well.”

**Reconsultation and certainty of effective treatment is important.** The wish for imminent and effective symptom relief was important and made UTI patients visit the FP. Thus, some patients mentioned an initial fear of prolonged suffering if trial drug wouldn’t work. In this situation, the FP’s demand to reconsult in case of persisting symptoms and the possibility to receive antibiotic treatment then promoted their decision to participate in the trial.

P16: “(…) and that if no improvement had occurred ... that I could report to my FP again and then initiate further treatment. That was something like an insurance for me.”

#### c) FP-patient-relationship

Here, aspects influencing trial participation arising from the FP-patient-relationship are considered. In some cases,** trust in the FP** promoted the decision to participate in the trial. Some patients seemed to be absolutely sure that the FP, or in one case the practice assistant, would never recommend the trial if it had been something bad or harmful, since the FP knows patients health best and the relationship is based on long-term confidence.

P14: “Yes, basically I have trust in my doctor. I feel in good hands. (…) And of course, they now have a leap of faith. No, therefore I had no concerns that they would try anything or that something bad would happen to me.”

The **communication** with the FP reassured some patients more than the information sheet – they highly valued the personal information and discussion of the trial which made them feel safe.

P6: “And he assured me that nothing bad happens, that the drugs that are used are not any old experimental laboratory drugs (…). I do not know if I would have readily decided [to participate] without a discussion with the FP.”

Even the FPs’ or practice assistants’ **personal conviction** that the trial was good and relevant convinced patients.

P8: “As a result, she [FP] had actually convinced me, because she was convinced of it [ICUTI trial] herself.”

In contrast, other participants decided trial participation completely on their own, based on the written information sheet.

P20: “Because I happened to read this poster (…). I had just seen it, and thought: it fits. And that’s why I raised it myself with the doctor. – (Did the doctor or nurse play a role?) – Nope. I had already decided for myself right from the outset. (...) I was immediately sure.”

Two interviewees didn’t even know the FP before since it was their first visit in the practice – and took part as well. In one case, thankfulness for the last minute-appointment led to participation.

P2: “And the nurse asked me to participate... and because I was glad I got to see someone at all, I naturally agreed.”

### 2) Why do patients refuse trial participation? Decliners reasons

**Concrete preferences:** Four decliners (D1, D2, D3, D5) had in common very firm convictions regarding (non) antibiotic treatment, varying from total decline of potential randomization to the antibiotic treatment arm to the wish for delayed antibiotic prescription.

D1: “And then I went [to the doctor] with the expectation that I will probably have to take an antibiotic. (…) And then he proposed a study (…) I said, I cannot participate in the study. I’d be happy to try the ibuprofen, and please prescribe me an antibiotic as well, just in case (…)”

Nevertheless, even the decliners expressed that they really approved the trial and found it important to find therapeutic alternatives to antibiotic treatment. Decliner 2,3 and 5 pointed out that they would have taken part in the trial if symptoms had been worse – in this case they would have accepted to be potentially randomized even to antibiotic treatment.

D2: “If I had had a massive urinary tract infection and had I said ‘I need something now’, then I would have participated.”

Due to the small number of interviews, further reasons to decline trial participation are described individually:

Decliner 1 had already been suffering quite a while, tried own medication, had a **strong desire to imminent pain relief** and demanded a (delayed) antibiotic prescription. **Additional effort by study procedures**, i.e. telephone interviews and eventual revisiting which did not fit into working hours was mentioned as a hampering factor as well.

D1: “The first thought on the word study was: Oh no, please not, I want pain relief now and I have no desire to experiment. (…)

Why I’ve decided against the study, because (…) if it does not work, then I will have to go and see a doctor again, which is always difficult to reconcile with work. (…) and the study will involve additional effort, with many meetings or phone calls.”

Decliner 2 had only minor complaints and did not want any strong drugs at all. She also pointed out that **randomization and blinding** was an additional barrier to participate.

D2: “And fundamentally I was not opposed, however (...) but when he told me that I will get a drug as part of this study (...) then I kept my distance. (...) because I was thinking: Well, you do not need drugs actually. Maybe just a homeopathic remedy or something like that to solve the whole problem.”

D2: “And that (...) was what really stopped me. I am willing to participate in studies (...) but if I do not know what I am taking then I am not so supportive.”

Decliner 3 wanted to treat herself as well with tea and homeopathics as long as symptoms were slight, but asked for an antibiotic prescription “for safety”, due to recent experiences with UTI.

D3: “Meanwhile, (…) I know that I can get things under control by drinking lots of tea and [taking] homeopathic stuff. But as a backup, I always like to have a prescription for an antibiotic at home.”

Contrastingly, refuser 4 refused after initial agreement, since she **feared severe health risks** after the emergency card was handed out. In her eyes the trial turned out to be potentially life-threatening, caused by the term “emergency card”.

D4: “initially I said yes (...) I would then have to have this emergency card. So that if something happened, so they knew what I had taken. Then (...). I panicked, no, I don’t want this, I don’t want it (...) Because I only know about these [emergency cards] from patients taking Marcumar or diabetics (…), and there it’s a matter of life and death.”

Again, decliner 5 rejected trial participation since she preferred non-antibiotic treatment. She also would have taken part if symptoms had been worse.

### 3) Barriers for trial participation from participants view

Being asked for potential barriers for other patients to participate in the trial, participants mentioned several aspects. Many stated that quite a number of patients may have **neither interest in nor feel an obligation for research**.

P13: “. .. because many think, yes give me the medication and I’ll go home and then just leave me in peace.”

In particular, **missing benefits or perceived disadvantages** in term of health risks were seen as a potential hindering factor for others. Trial participation could imply delayed symptom relief or involve a risk of worsening which does not fit into patients’ expectations.

P8: “(…) just that people who are sick anyway, do not want to risk worsening of their condition (…) you go to the doctor because you want to make it better.”

The uncertainty whether the trial drug is effective or not, and the fear of being reduced to a test object may also be perceived as a disadvantage.

P15: “fear (…) that one would be subjected to some kind of medication (... ) that you would somehow be used as a guinea pig.”

Some participants assumed that trial-related **time effort** may keep patients from participating, which holds particularly true for employed patients.

P13: “with more complex, time-consuming studies, they simply do not have the time to attend”

Further, the **influence of media** reporting negatively about trials was addressed as potential barrier.

P20: “this is also quite a large theme in the media, because it is always portrayed as very negative, or immediately some kind of drama is made out of it”

## Discussion

Results show that patients presenting in family practices with UTI symptoms participated in a double-blind randomized-controlled drug trial for various, partly overlapping reasons. A relevant trial theme and perceived personal benefit in terms of “better” treatment for recurrent UTI promotes participation as well as – more generally – the wish to support research and to help others. 

Furthermore, to feel safe since trial participation is not associated with disadvantages, for example regarding symptom relief, reassures patients significantly.

In some cases, trust in the FP plays an important role in the decision process. In contrast, some patients show strong individual treatment preferences and therefore decline trial participation. 

In many respects, these findings correspond to those of comparable studies with cancer or chronically ill patients’ motivation to participate in trials. A patient-relevant trial theme is known to be an important driver for participation [7], [13]. Many UTI patients, in particular those affected by recurrent UTI, reject repeated antibiotic treatment and are open towards alternative treatment strategies. In a questionnaire study with female UTI patients, Willems et al. revealed that 66% would agree to postpone antibiotic treatment and 88% of patients know about disadvantages of antibiotic treatment [17], [20]. Thus, the ICUTI trial seems to have met patients’ original research needs, which may have promoted participation in many cases.

Patients with acute conditions often show strong preferences towards their favored treatment, well-known from previous disease episodes. Understandably, the wish for soon and effective symptom relief is prior – but may lead to decline participation. Thus, these patients need to be offered effective trial drugs or at least an alternative treatment if the trial drug does not work. In our study, many interviewees confirmed that they felt reassured since both trial drugs were known as effective and FPs initially advised reconsultation in case of persisting symptoms. 

In contrast, for patients with cancer or severe chronic conditions treatment options are often limited. Against this background, a trial may be perceived as a major chance for recovery or healing. Thus, for these patients the decision to participate in a trial might be considerably more driven by hope, desire and trust [21]. Even in cancer patients, Jenkins et al. showed a significantly higher acceptance rate for trials with active treatment in every arm in a questionnaire study [12].

To integrate patient preferences in the trial participation progress, Mills et al. trained recruiters to address patients’ treatment preferences clearly to motivate even those who did not have considered trial participation before [22]. Other trialists meet patient preferences by integrating a patient preference arm in the trial, as done by McCann et al. in the REFLUX trial [13].

On the other hand, UTI patients are often affected by recurrences, which makes UTI resemble to a chronic disorder and encourages at least some patients to support further research – in order to benefit personally, not only from the favored non-antibiotic treatment, but also from the option of self-treatment without FP consultation. Comparably, patients with chronic conditions appreciate personal benefit from trial participation. Several studies could show that decisions for participation depends not only on altruism in terms of willingness to help others, but also on perceiving personal benefits (or no disadvantages) [13], [23]. Similarly, Madsen et al. showed in a sample of cancer patients that research may be perceived as important, but as soon as patients are involved personally, personal benefits are prior [11]. Thus, the decision to participate in a trial implies a complex process, characterized by weighing up personal benefits and disadvantages [6], [9], [21]. 

The process of randomization and clinical equipoise is often hard to understand for patients [11], [12]. Yet, in trials with two accepted treatment strategies this seems to be less important as it might be in placebo-controlled trials. Only one of our interviewees mentioned randomization as a problem. However, “feeling like a test object” was brought up as potential hampering factor, possibly rooted in lack of knowledge about ethical principles in modern clinical research and should be considered in future.

Trust in the doctor is known to be a relevant motivational factor [11], [12], [21]. Although one could expect the FP to be considered a trustworthy advisor, our results are not consistent: even if for many patients, the FPs recommendation to participate was important, others decided completely on their own about trial participation.

Interestingly, only one of our participants appreciated explicitly the aspect of patient-centred research in the ICUTI trial, contrasting to usual commercialized development in the health system. It seems as if this fact could be promoted more, both to sharpen patients’ perception of research procedures and to motivate for trial participation.

## Limitations

The constant critical attitude of our interviewees toward antibiotic treatment shows that UTI patients with a strong desire for an alternative treatment are probably more likely to participate both in ICUTI and the interview study, resulting in an inevitable inclusion bias and, potentially, in socially desirable answers during interviews. In particular, due to the low number of decliner interviews, the presented decliner aspects express more an picture detail than a complete view of their attitudes. As mentioned by participants, many patients might not take part in trials due to a lack of interest and perceived disadvantages. It is known that these patients attitude can hardly be assessed, because they tend to be reluctant to be interviewed as well and do not like to talk about their negative attitudes [21].

As for our study, patient recruitment for ICUTI was absolutely prior, and over long months all efforts aimed at motivating practices to recruit in ICUTI. Thus, we wanted to avoid additional strain with repeated inquiries to recruit decliners in this embedded study. 

## Conclusions

Results of this interview study show that many findings from earlier studies regarding trial participation of patients with cancer and chronic conditions can be transferred to the primary care research setting. To make use of these results for further clinical trials about acute and common conditions in family medicine, we captured the following issues i.e. for patient information sheets, or for FP/staff training when discussing trial participation.

- Emphasize personal benefits for patients – these are relevant drivers and should be demonstrated clearly, i.e. in written/oral trial information
- Feature patient relevant trial themes
- Provide a maximum of objective and subjective safety, i.e. regarding effective symptom relief – this facilitates patients’ decision to participate
- Declare patients’ trial efforts clearly, and keep it as comfortable as possible
- Highlight all patient-centred aspects in your trial
- Acknowledge that patients’ willingness to participate in a comparative effectiveness trial with two active arms and well-known drugs is likely to be higher than in placebo-controlled trials
- Inform patients about medical research, need for evidence, trials and basic ethical aspects whenever possible
- … that means for trialists: consider these aspects early enough, and if possible arrange a preceding trial discussion with potential patients to catch their views towards the planned intervention and procedures.

## Abbreviations

RCT: randomized-controlled trial

UTI: urinary tract infection

FP: family practitioner

ICUTI: Immediate versus conditional antibiotic treatment of uncomplicated urinary tract infection

BMBF: Bundesministerium für Bildung und Forschung (German Ministry for Research and Education)

ICH-GCP: International Conference of Harmonization, Good Clinical Practice

## Notes

### Competing interests

The authors declare that they have no competing interests.

### Authors’ contributions

JB, IG and EHP had the original idea and conceived the study together with MLD. JB, SB, IG and MLD developed the interview guideline. SB performed the interviews, supervised by JB and MLD. JB and SB analyzed the data and discussed it regularly with MLD. Final results were discussed with all authors. JB drafted the manuscript with the contribution of IG, MLD and EHP. All authors read and approved the final manuscript.

### Acknowledgements

The authors would like to thank all patients who were willing to participate in the interviews, in particular those who declined trial participation. Further we thank all staff members for supporting this study.

## Figures and Tables

**Table 1:**
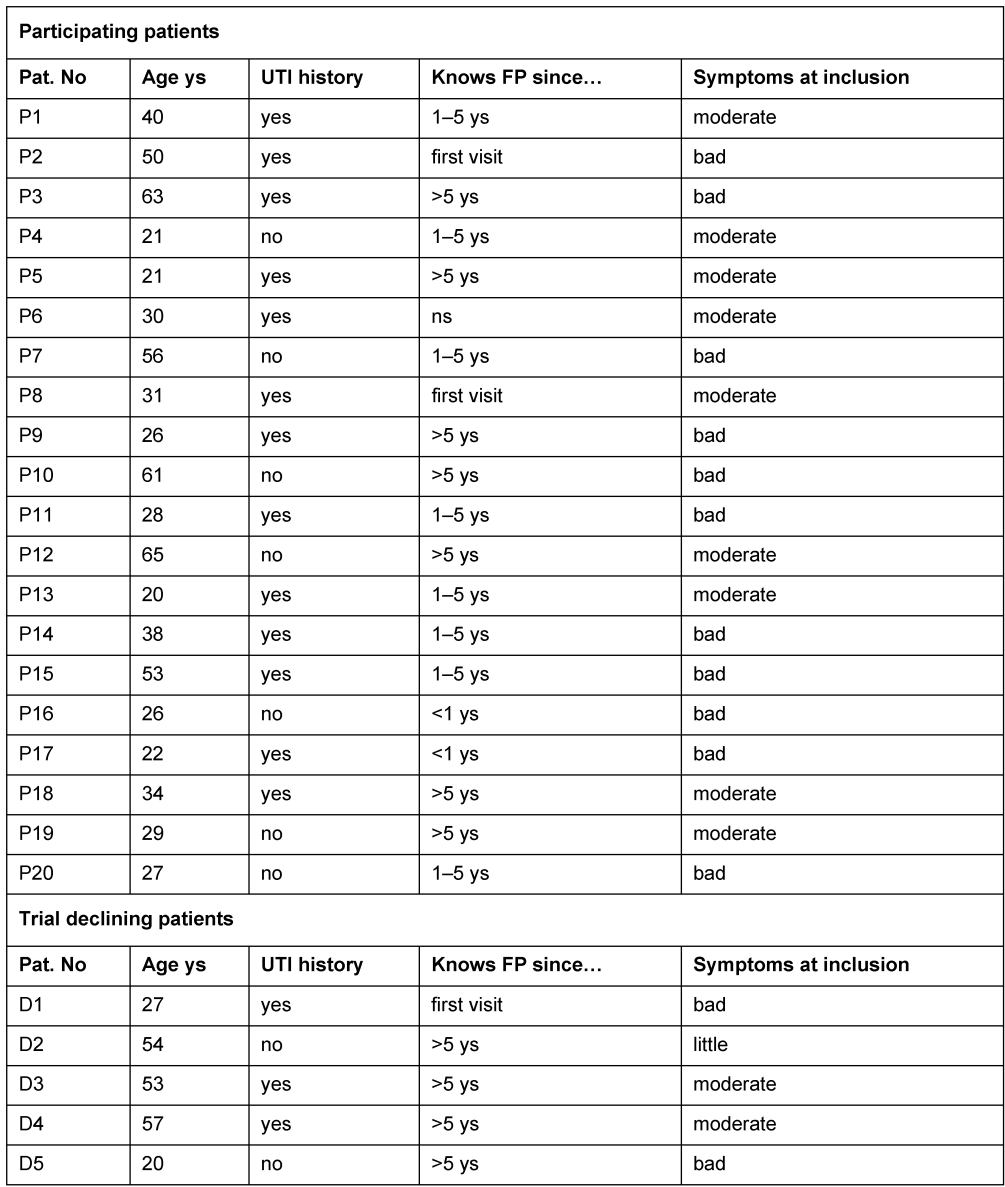
Characteristics of interview partners
